# Rice *stripe1-2* and *stripe1-3* Mutants Encoding the Small Subunit of Ribonucleotide Reductase Are Temperature Sensitive and Are Required for Chlorophyll Biosynthesis

**DOI:** 10.1371/journal.pone.0130172

**Published:** 2015-06-23

**Authors:** Xiaoqiong Chen, Ling Zhu, Long Xin, Kangxi Du, Xiuhua Ran, Xiaoyun Cui, Quanju Xiang, Hongyu Zhang, Peizhou Xu, Xianjun Wu

**Affiliations:** 1 Rice Research Institute of Sichuan Agricultural University, Key Laboratory of Southwest Crop Genetic Resources and Improvement Ministry of Education, 211 Huimin Road, 611130 Wenjiang, Sichuan, China; 2 College of Resources, Sichuan Agricultural University, 211 Huimin Road, 611130 Wenjiang, Sichuan, China; China National Rice Research Institute, CHINA

## Abstract

We induced mutants, *stripe1-2* (*st1-2*) and *stripe1-3 *(*st1-3*), from rice *(Oryza sativa* L.) *Indica* 9311 using Ethyl methanesulfonate (EMS). Both *st1-2* and *st1-3* mutants encoded the small subunit of ribonucleotide reductase 1 (RNRS1), differed in the location of the mutated base, and displayed white-stripe from the L2 stage through maturity. The mutants were sensitive to temperature, and their chlorophyll content increased with the increase in temperature; however, they did not revert to normal green leaf phenotype under field conditions. The mutant *st1-2* showed loosely arranged thylakoid lamellar structure as compared with wild-type (WT) plants. Contrastingly, *st1-3* displayed normal thylakoid lamellar structure, good agronomic traits, and higher yield than *st1-2* but lower yield than WT. Three-dimensional structure prediction for RNRS1 indicated that the mutation in Val-171 residue in *st1-2* influenced the connection of RNRS1 to iron, causing abnormal development of chloroplasts. Real-time PCR analysis showed that the expression levels associated with chlorophyll biosynthetic pathway and photosynthesis were affected in *st1-2* and *st1-3* at different temperatures and different developmental stages.

## Introduction

Plastid and nuclear genes regulate the transformation of proplastids to photosynthetically active chloroplasts during plastid development. Chloroplast protein in the plant cell is mainly encoded by nuclear genes while less than 5% is encoded by cytoplasmic genes [1, 2]. Mutations in these genes or chloroplast associated mutations cause leaf discoloration and affect seedling viability. The resulting mutants have diverse phenotypes and are variously referred to as virescent (*v*), stripe (*st*), albino, chlorina, zebra, and yellow variegated [3]. Variegation/stripe mutants associated with chlorophyll (Chl) and chloroplast were identified in various plant species including Arabidopsis [4–6], maize [7] and tomato [4]. Iba et al. and Yoo et al. cloned Virescent and stripe mutants *v1*, *v2*, *v3*, and *st1* from rice [8, 9]. The *v1* gene encodes a chloroplast localized protein NUS1, which is involved in the activation of plastid genes; it promotes the establishment of the plastid genetic system during early development in rice [10,11]. The *v2* gene encodes a novel plastid and mitochondria-localized guanylate kinase that function in chloroplast differentiation [12,13]. The *v3* gene encodes the large subunit of ribonucleotide reductase (RNRL) that possesses binding sites for substrate and allosteric effectors. Mutant leaves show chlorosis from the L2 stage and contains only a trace amount of chlorophyll in their leaves. Moreover, the *st1* gene encodes the small subunit of ribonucleotide reductase 1 (RNRS1), involved in DNA synthesis and repair during early leaf development [9]. RNRS1 is located in the nucleus and undergoes a nucleus to cytoplasm redistribution for continued biosynthesis of deoxynucleotides during DNA replication or repair [14]. RNRS1 mutants exhibit chlorotic leaf from L2 at the 20°C. The leaves of *v* mutant plants suffer from Chl deficiency, which develops during the early growth stages at temperature 20°C to 30°C; however, the leaves show normal green color below 20°C or above 30°C during later growth stages [15]. Both *St1-*1 and *v3* are similar in temperature sensitivity; *v3* is the stronger phenotype of the two. On the other hand, *st1-2* mutant exhibited longitudinal white stripes throughout entire development phase from L2 and showed chlorosis until maturity despite a slight increase in their chlorophyll content. Rice *st1-3* mutants were chlorotic from L2 through maturity but produced normal leaves in paddy fields at 30°C. The mutants *v1*, *v2*, *v3* and *st1* show variable phenotypes for leaf color corresponding to temperature changes [9–13]. However, *st1-2* and *st1-3* differ significantly in their sensitivity to temperature because they harbor mutations at different loci. The genes *OsHAP3A*, *OsHAP3B*, *OsRpoTP*, *OsRpoTm*, *OsPPR1*, and *Ostrxm* regulate chloroplast biogenesis [16–19]. Mutations in the large and small subunits of ribonucleotide reductase (RNR) cause white-stripe leaf in rice [9]. Moreover, most genes affecting chlorophyll content are photosynthetic genes encoded in the plastid (*psbA*) and nucleus (Lhcp*II and RbcS*) among which the plastid genes encode the transcription/translation apparatus (*rpoA*, *rpoB*, and *rps7*) [9–13]. However, direct involvement of RNRS1 in chlorophyll biosynthesis is less understood. Here, we report the genes involved in chlorophyll biosynthetic pathway, which were affected because of a mutation in RNRS1.

## Materials and Methods

### Plant Materials and growth conditions

Rice *(Oryza sativa* L.) *st1-2* and *st1-3* mutants having retarded growth rate were induced from *Indica* 9311 by ethyl methanesulfonate (EMS). The mutants were planted in a paddy field in April to September in Wenjiang District (latitude 30°429N, longitude 103°509E, altitude 539.3 m), Chengdu City, Sichuan Province on privately owned land with the owner’s permission. The collection of the samples did not involve endangered or protected species, and was safe to the environment.

### Transmission electron microscopy analysis

Rice leaf samples of 9311 from mutants, *st1-2*, and *st1-3* were prefixed in 3% glutaraldehyde, postfixed in 1% osmium tetroxide, dehydrated in acetone series, infiltrated in Epox 812 and embedded. The semi-thin sections were stained with methylene blue, and the ultra-thin sections were cut with a diamond knife and stained with uranyl acetate and lead citrate. Sections were examined under a Transmission Electron Microscope (TEM; HITACHI, H-600IV, Japan).

### Measurement of photosynthetic pigments

Chls extractions were carried out at tillering, jointing, and heading stages. Rice leaf tissues (0.2 g) were soaked in ice-cold 80% (v/v) acetone in the dark, and sequentially extracted for 48 h at room temperature with shaking 80 rpm/min. Then residual plant debris was removed by centrifugation, and the supernatants analyzed with a visible spectrophotometer UV-1800 (Shanghai. China). Chls and carotenoids were measured at 663 nm, 646 nm, and 470 nm and determined as described by Lichtenthaler [20].

### Measurement of photosynthesis

Leaf photosynthesis was measured for 15 lines. To ensure that the leaves measured were similar in age, and developmental stage, only the flag leaf of each plant was sampled from 15 randomly selected lines. The measurements in each stage were roughly taken between from 9:30 to 12:00 AM. Measurements were made using a portable gas exchange system LI-COR 6400 PSC 2934 (LI-COR Inc., Lincoln, NE, USA). Light levels at each time point were set to reflect the red, blue light source, 1000 μmol m^-2^s^-1^. Photosynthesis measurements were recorded at a steady state.

### Map-based cloning of *St1-2* and *St1-3*

For map-based cloning of the *st1-2* gene, the mapping population consisted of 1090 F2 generated by a cross between *st1-2* and *Japonica* 02428 (*Pangxiegu* × *Jibanggu*). For the fine mapping of the *st1-2* locus, 1253 F3 recessive lines were further generated from F2 heterozygous lines progeny. The *st1-3* mapping population, which consisted of 700 F2 individuals, was generated by the cross between *st1-3* and 02428. For the fine mapping of the *st1-3* locus, 900 recessive lines were further generated from F2 heterozygous lines. The SSR markers were obtained from GRAMENE (http://www.Gramene.org/bd/markers/ and the Arizona.edu/fpc/rice) websites, and were used to construct linkage maps as described by McCouch et al. (2002). To narrow down the region of the target locus, we conducted a Blast search using the NCBI database. Insertion/deletion (InDel) sequence divergence between Japonica (Nipponbare) and Indica (9311) were determined by this locus (http://www.Ncbi.nlm.nih.gov/BLAST/). InDel markers were developed around the sequence divergence including 15 to 100 bp InDel using the Primer 5.0 software. The allelic tests were performed by reciprocal cross between *st1-2* and *st1-3*.

### Sequence analysis and prediction of protein structure

The full-length DNA and protein sequence of *st1-2* and *st1-3* and their homologs were retrieved from GeneBank (http://www.ncbi.nlm.nih.gov). The chloroplast signal peptide was predicted by the SignalP 4.1 Server [21]. The three-dimensional structure was predicted using the SWISS-MODEL (http://swissmodel.expasy.org/) using human ribonucleotide reductase (PDB id: 3hf1; 68.13% identity) as a template. The images were generated using PyMOL (http://pymol.en.uptodown.com/).

### Temperature treatments

Rice 9311 *(Oryza sativa* L.), *st1-2-* and *st1-3* mutants were grown using potting soil in a reach-in model Growth Chamber Reach ZBX100GS, and at 26°C /14h of light, 22°C/ 10h of dark, and planted time for 30 d. The plants were transferred to a controlled environment growth cabinet, arranged randomly within the cabinet and spaced to prevent shading. Plants were grown in 14 h photoperiods (6660 lx) and 10 h dark, at a constant 75% humidity for a period of 6 d. The plants were subject to different temperature treatments, and were designated as: L20 / D16 (light 20°C / dark 16°C), L26 / D22 (light 26°C / dark 22°C), L30 / D26 (light 30°C / dark 26°C), and L30/D24 (light 30°C for 24 h). RNA was extracted at the end of the treatment period from samples with three biological replicates.

### Real-time reverse transcription-qPCR analysis

Total RNA was extracted using a Trizol RNA mini-kit following the manufacturer’s protocol (Roche, Mannheim, Germany). cDNA was synthesized from 1 μg of total RNA using the iScriptcDNA Synthesis kit (Bio-Rad). qPCR was performed using the CFX96 Real-Time System (BIO-RAD Inc. CA, USA), following the manufacturer’s instructions. All reactions were performed with Fast Start Universal SYBR Green Master (ROX) (Roche, Mannheim, Germany), according to the manufacturer’s instructions. PCR reactions were performed in triplicate (2 replicates per reaction) and were averaged using 5 μl of SsoAdvanced SYBR Green Supermix: 0.3 μM of each primer, 12.5 ng cDNA, and nuclease-free water to a final volume of 10 μl. A negative control (water) was included in each run. Reactions were incubated at 95°C for 10 min, followed by 39 cycles of amplification at 95°C for 10 s and then 60°C for 30 s, after which a final extension step was performed at 72°C for 1 min. Fluorescence was measured at the end of each extension step. PCR amplification was followed by melting curve analysis with continual fluorescence data acquisition during the 65°C to 95°C melt. The raw data were analyzed with CFX Manager Software and expression was normalized to that of the rice actin1 gene (forward primer: CTTCATAGGAATGGAAGCTGCGGGTA; reverse primer: CGACCACCTTGATCTTCATGCTGCTA) to minimize variation in cDNA template levels. Relative expression levels were calculated using the comparative threshold (Ct, cycle value) method. Mean values were obtained from three biological replicates, each determined in triplicate.

## Results

### Phenotypic characterization of *st1-2* and *st1-3* mutant

**Fig 1:**
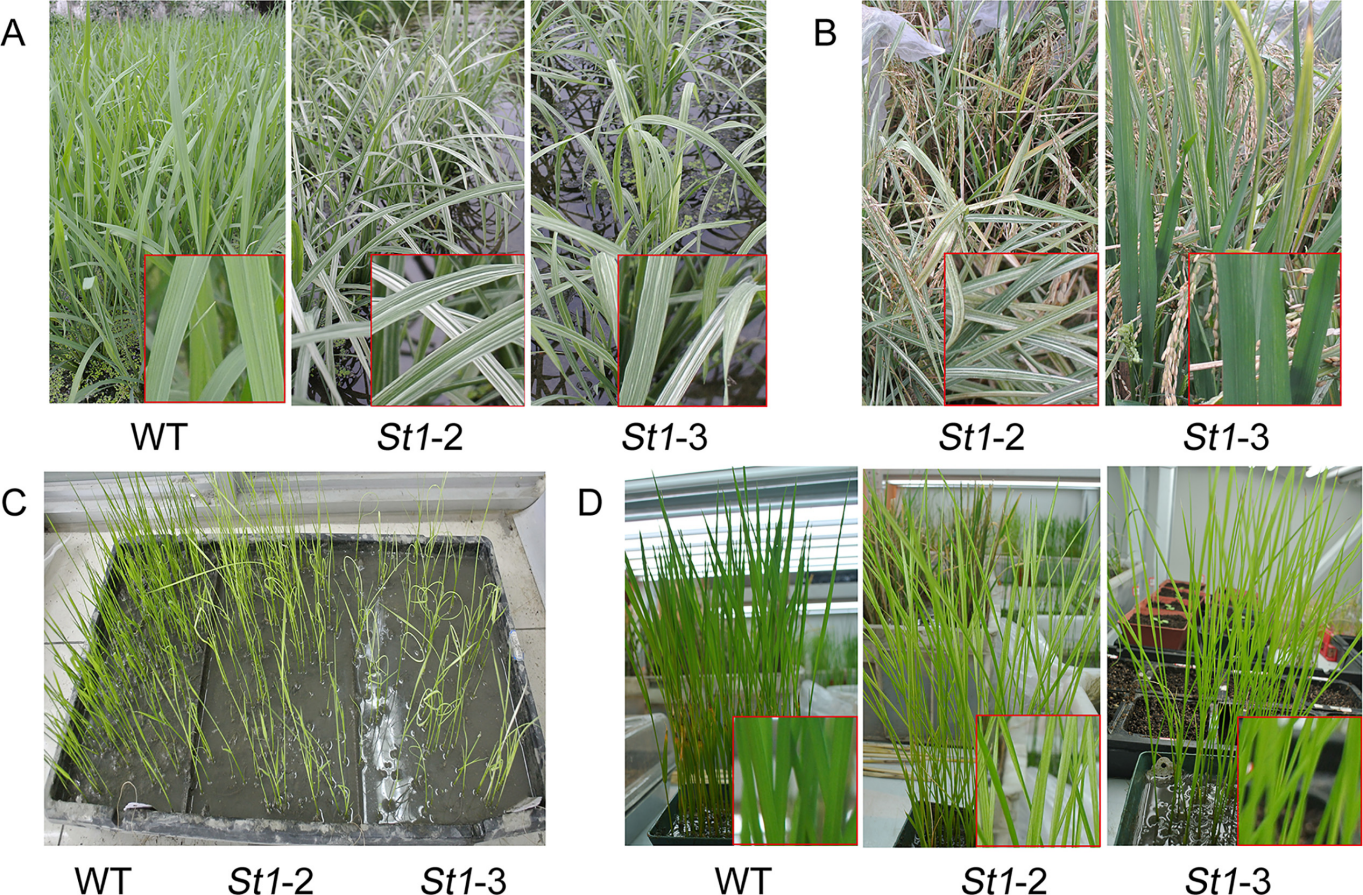
Phenotypic characteristics of WT, *st1-2* and *st1-3* mutants. A, Phenotypes of wild-type and mutant rice during the tillering stage in a paddy field. WT, Wild-type *indica* 9311. *St1-2* and *st1-3* were mutants derived from *Indica* 9311 induced by ethyl methanesulfonate (EMS). B, Phenotypes characteristics of mutant rice at the mature stage in a paddy field. C, Leaf phenotypes of wild-type and mutant rice during the second leaf stage in the greenhouse cabinet. D, Leaf phenotypes of wild-type and mutant rice during the third leaf stage in plants grown in the potting soil of greenhouse.

*st1-2* and *st1-3* plants exhibited normal green leaves similar to those of the wild-type before L2 under paddy field conditions. Post L2, both *st1-2*, and *st1-3* showed leaf chlorosis until the tillering stage (Fig 1A). The leaves showed white stripes on a green base from tillering until maturity (Fig 1B). The mutant *st1-2* showed more conspicuous white stripes on leaves than *st1-3* from the maximum tillering stage. The white stripes on leaves are prominent until maturity and could not recover back to complete green color with an increase in temperature (Fig 1C). Contrastingly, *st1-3* leaves recovered faster to produce chlorophyll with an increase in temperature and recovered to their normal green color at 30°C. Furthermore, *st1-2* leaves are small, short, and narrow as compared to those of wild-type plants. On the other hand, *st1-3* leaves did not show any changes in morphology other than the white stripe phenomenon (Fig 1B). Our observations are consistent with the results of Yoo et al. [9] and indicate that the mutant phenotypes are expressed in a growth stage-dependent manner and differentiate during the regular growing season. Moreover, *st1-2* significantly influenced the growth of the plant as compared with *st1-3*.

### Observation of chloroplast development by electron microscopy

**Fig 2:**
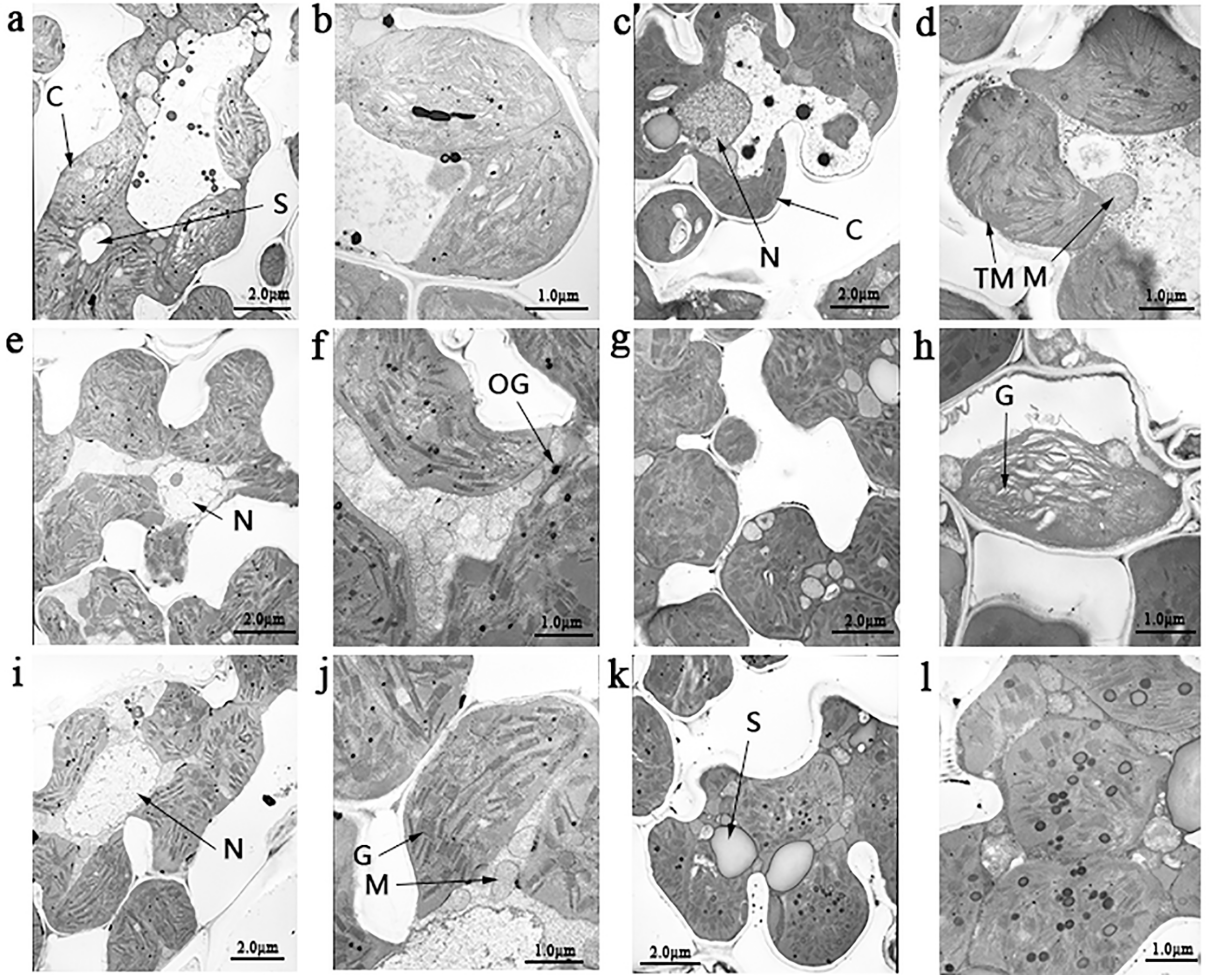
Ultrastructure of chloroplasts in the mesophyll cells of mutant plants. a, shown are transmission electron microscopy images of chloroplast of the wild type (WT) in 5000 times in tillering stage; b, enlarged in 20000 times for WT in tillering stage; C, 5000 times for WT in jointing stage; d, 20000 times for WT in jointing stage; e, *st1-2* at 5000 times in tillering stage; f, *st1-2* at 20000 times in tillering stage; g, *st1-2* at 5000 times in jointing stage; h, *st1-2* at 20000 times in jointing stage; i, *st1-3* at 5000 times in tillering stage; j, *st1-3* at 20000 times in tillering stage; k, *st1-3* at 5000 times in jointing stage; l, *st1-3* at 20000 times in jointing stage; C, chloroplast; F, fat; G, grana; M, mitochondria; N, nucleus; OG, osmiophilic plastoglobuli; S, starch granule; TM, thylakoid membranes.

Ultrastructural studies showed that WT chloroplasts had a considerable number of starch granules as compared with *st1-*2 and *st1-*3 in the jointing stage (Fig 2). However, the chloroplast of *st1-*2 and *st1-*3 showed well-developed lamellar structures with regularly stacked grana and thylakoid membranes. There was no significant difference in the number of chloroplasts per cell between wild type and mutants. In the heading stage, the thylakoid structure of *st1-*2 was loosely arranged as compared with the wild type, but *st1-3* did not any show significant differences compared with the wild-type plants. Both, *st1-*2 and *st1-*3, showed a greater number of osmiophilic acid particles than WT in the jointing and heading stages. These results indicate that *st1-*2 and *st1-*3 affect starch synthesis because of abnormal chloroplast development.

### Chlorophyll and carotenoid content of WT and mutants

**Fig 3:**
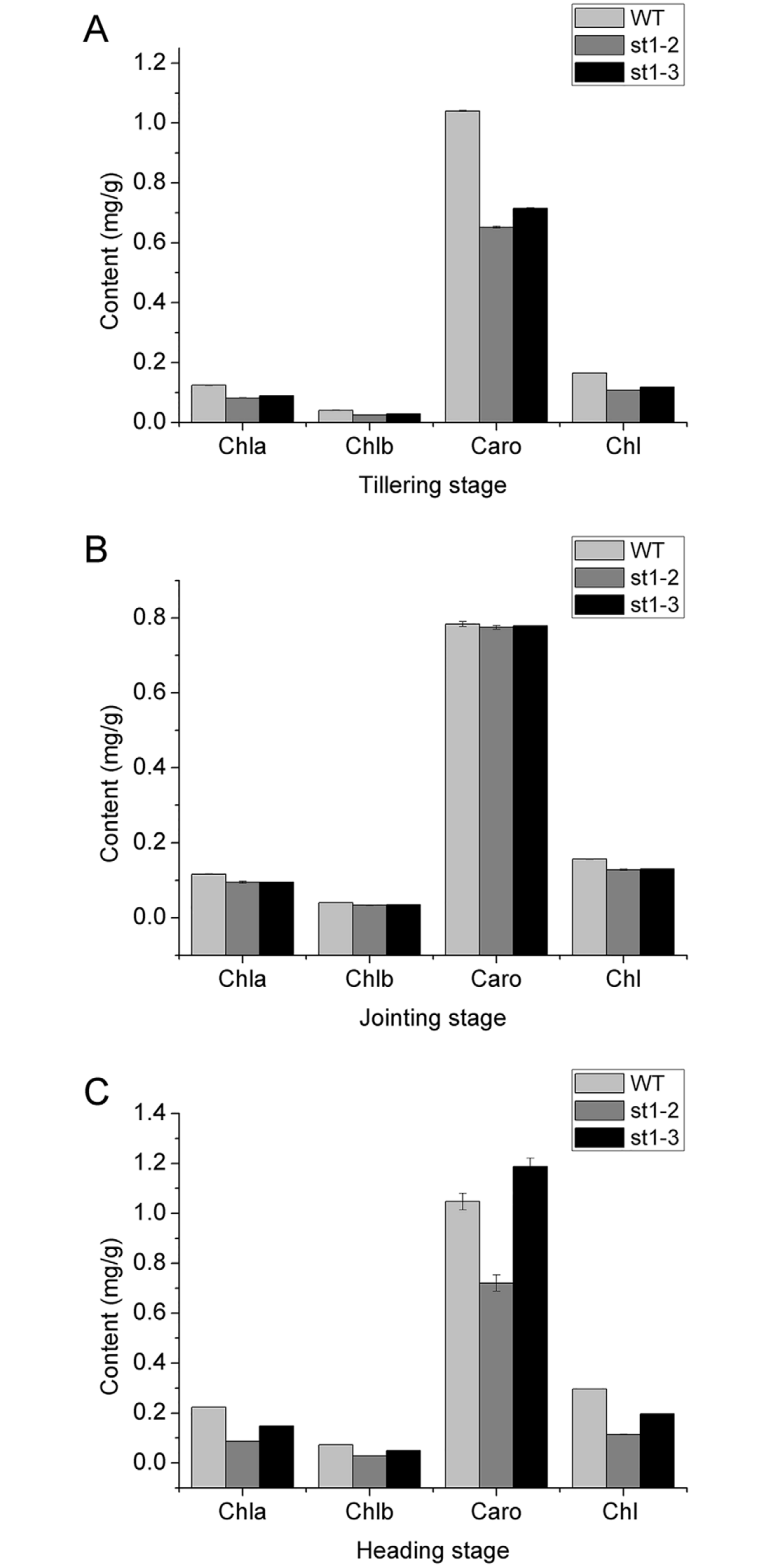
Chlorophyll and carotenoid contents of WT, *st1-2* and *st1-3* in different developmental stages. Chl *a*, chlorophyll a; Chl *b*, chlorophyll *b*; Caro, carotenoid; Chl, total chlorophyll content.

Chl *a* content of WT was significant higher than that of mutants in all stages of development. Chl *b* content of WT was only slightly higher than that of mutants in the heading stage and was not different from mutants in the other stages (Fig 3). Therefore, we speculate that the white-stripe mutants mainly regulate the gene encoding chl a in the chloroplast biosynthetic pathway. Chl *a* and *b* content of *st1-*3 was higher than that of *st1-*2 in both tillering and heading stages. There were no significant differences in chl *a* and *b*, and carotenoid content of *st1-2*, *st1-3*, and WT in the jointing stage. The mutants, *st1-*2, and *st1-*3, showed different agronomical parameters at different stages maintained at different temperatures. This led us to speculate that their differences are related to differences in their temperature sensitivities. Moreover, *st1-*3 was more sensitive to temperature than *st1-*2, although neither of the mutants reverted to normal from the white stripe phenotype with increasing temperature. Our study showed that *st1-2* is different from *v3*, and *st1* and *st1-3* mutants all of which exhibit normal phenotypes after heading (Fig 1B); thus, corroborating the results of Yoo et al. [9].

### Analysis of photosynthesis and agronomic traits in WT and mutant rice plants

**Fig 4:**
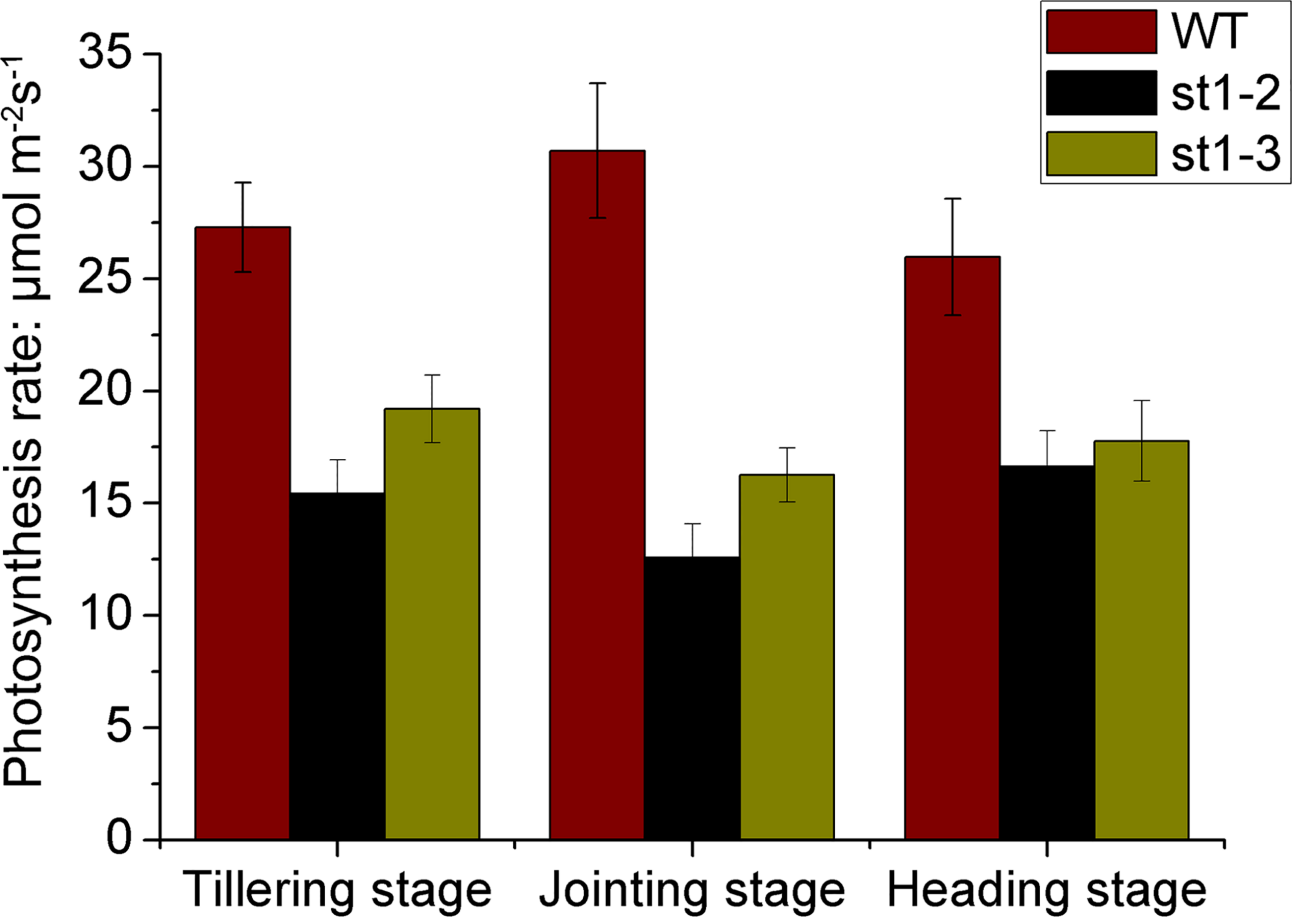
Photosynthesis rate of WT, *st1-2* and *st1-3* in different development stage.

The net photosynthesis rate was the highest in WT followed by *st1-3* and *st1-2* in all the three developmental stages (Fig 4). Agronomic traits such as plant height, number of tillers, numbers of spikelets, panicle length, 1000-grain weight, and setting rate of the mutants, *st1-3* and *st1-2*, significantly differed from those of WT (Table 1). The 1000-grain weight and setting rate was lower in *st1-2* compared with *st1-3*.

**Table 1:** Comparison of agronomic traits between *mutants* and wild type.

Traits	Wild-type	Mutants		Compared with WT (%)	
	(WT)	*St1-2*	*St1-3*	*St1-2*	*St1-3*
Plant height (cm)	119.9±1.28	101**±1.93	95.6**±2.75	-15.76	-20.26
Panicle length(cm)	22.34±0.63	21.76±0.71	21.46±2.87	-2.60	-3.94
No. of effective tillers per plant	8.80±1.20	8.60±1.41	8.20±1.30	-2.27	-6.82
Numbers of spikelets	234.2±50.04	140.1**±31.8	162.4**±38.83	-40.18	-30.66
Setting rate (%)	91.02±1.92	83.58*±1.92	90.4±3.02	-8.17	-0.68
1000-grain weight	29.16±1.28	23.41**±0.55	23.80**±0.97	-19.72	-18.38

Notes: ** and * mean significant at 1% and 5% level respectively compared with WT.

### Map-based cloning of *st1-2* and *st1-3* genes

**Fig 5:**
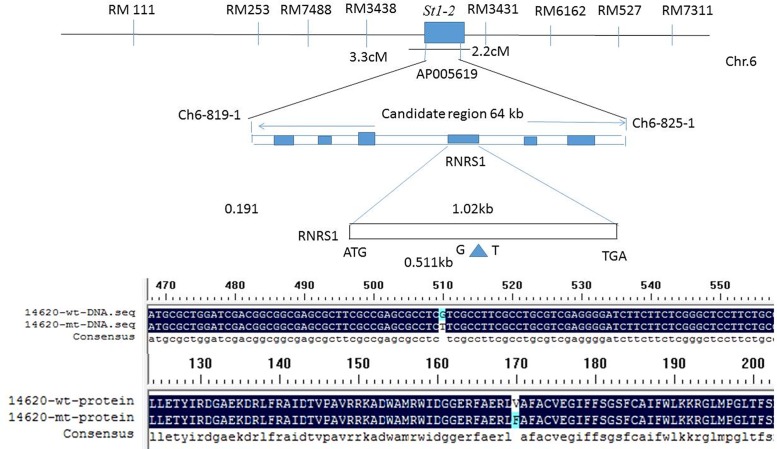
Fine mapping of *st1-2* gene (A) and alignment of DNA (B) and protein (C) sequences between WT and mutant. A, Genetic mapping of the *st1-2* gene using SSR and InDel markers. The locus of *st1-2* was initially mapped on the short arm of chromosome 6 between a 3.3-centimorgan (cM) to RM3438, and a 2.2-cM to RM3431 markers. B, Comparison of DNA sequences between mutant and the wild type. C, Comparison of protein sequences between *st1-*2 and the wild type.

We mapped the *st1-*2 gene from F1 to F3 plants that were generated from a cross between *st1-*2 (*indica*) mutant and 02428 (*Japonica*). Our results showed that the leaves of F1 plants were similar to WT. The white stripe in F2 plants was controlled by a single recessive gene, and the *st1-*2 locus was initially mapped using two simple sequence repeat (SSR) markers between RM3438 and RM3431, on the short arm of chromosome 6 (Fig 5A). We generated a fine physical map and developed InDel markers around the sequence divergence including 15 to 100 bp InDel using 26 primer pairs. *St1-*2 locus was finally narrowed to a 64-kb interval between Ch6-819-1 and Ch6-825-1, and on the sequence-tagged site AP005619 (Genbank accession number; Fig 5A). Four of 13 candidate genes possessed a function in this region in the Rice Genome Research Program. RT-PCR and DNA sequencing of four expressed genes in the *st1-*2 mutant revealed that the RNA small submit gene (designed RNRS1; LOC_ Os06g14620) comprised of only one exon, and a signal-base change (G511T) (Fig 5B) occurred in the *st1-*2 allele, which caused a missense mutation, Val to Phe (Fig 5C) as compared with that of wild. The *st1-3* gene was mapped using the F2 progeny of *st1-3* (*indica*) mutant crossed with 02428 (*Japonica*). Using PCR-based markers, the *st1-3* locus was initially mapped as *st1-2* SSR primers and was checked in a manner similar to *st1-2*. Our results showed that *st1-3* also was an allele of *st1-2*, but its locus was different from *st1-2*, which caused the C685T (Leu to Phe) mutation (S1 Fig). We further tested the alleles by a doing a reciprocal cross between *st1-2* and *st1-3*. The results also showed that *st1-2* was an allele of *st1-3* (S2 Fig).

### Protein structure analysis

**Fig 6:**
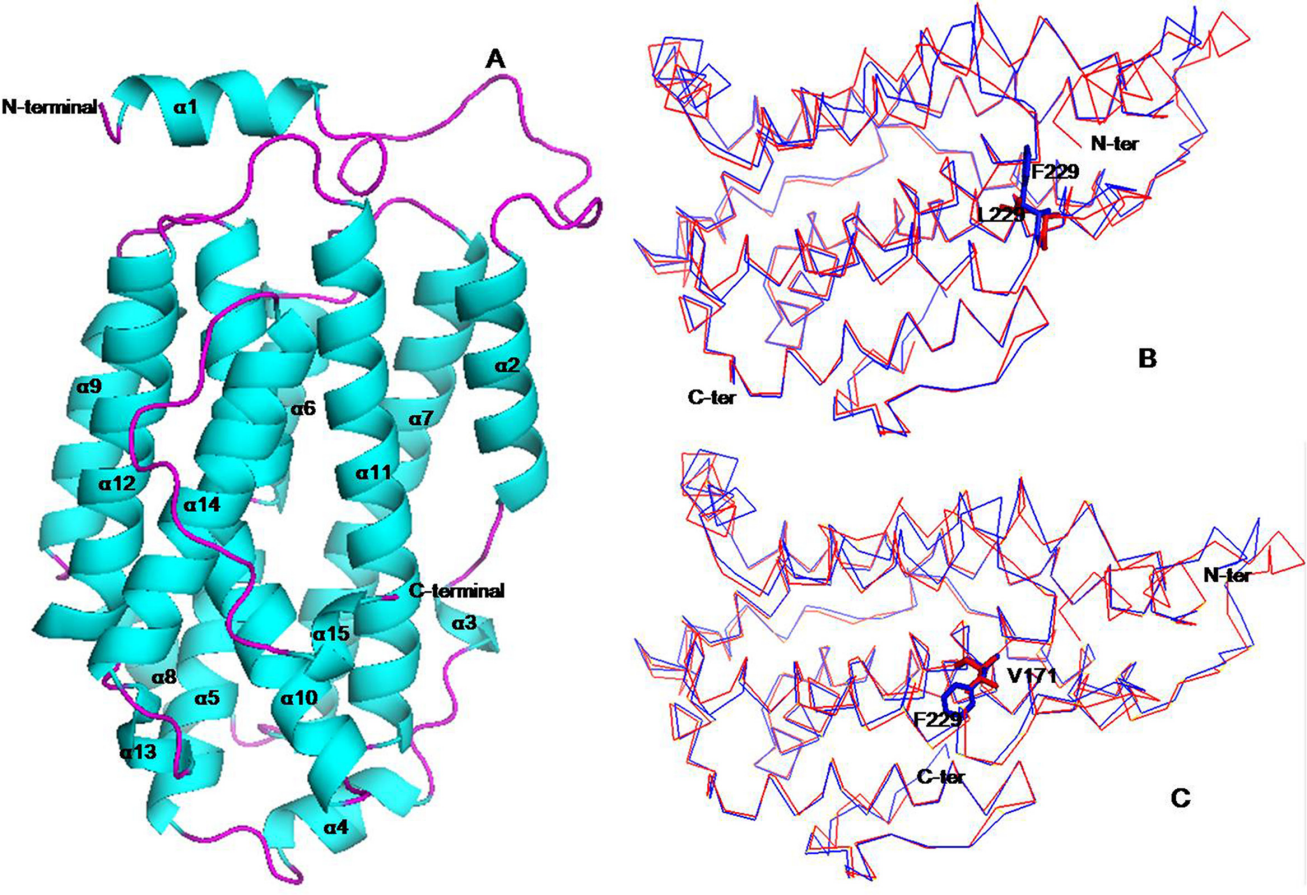
The putative tertiary structure of small subunit of ribonucleotide reductase 1. (A) Cartoon representation of RNRS1 molecular structure. (B) Comparison of the overall structure between RNRS1 (red) and *st1-2* (Val171 mutation, blue) and (C) *st1-3* (Leu229 mutation, blue). All figures were prepared using PyMOL. The mutation sites were represented by sticks.

**Fig 7:**
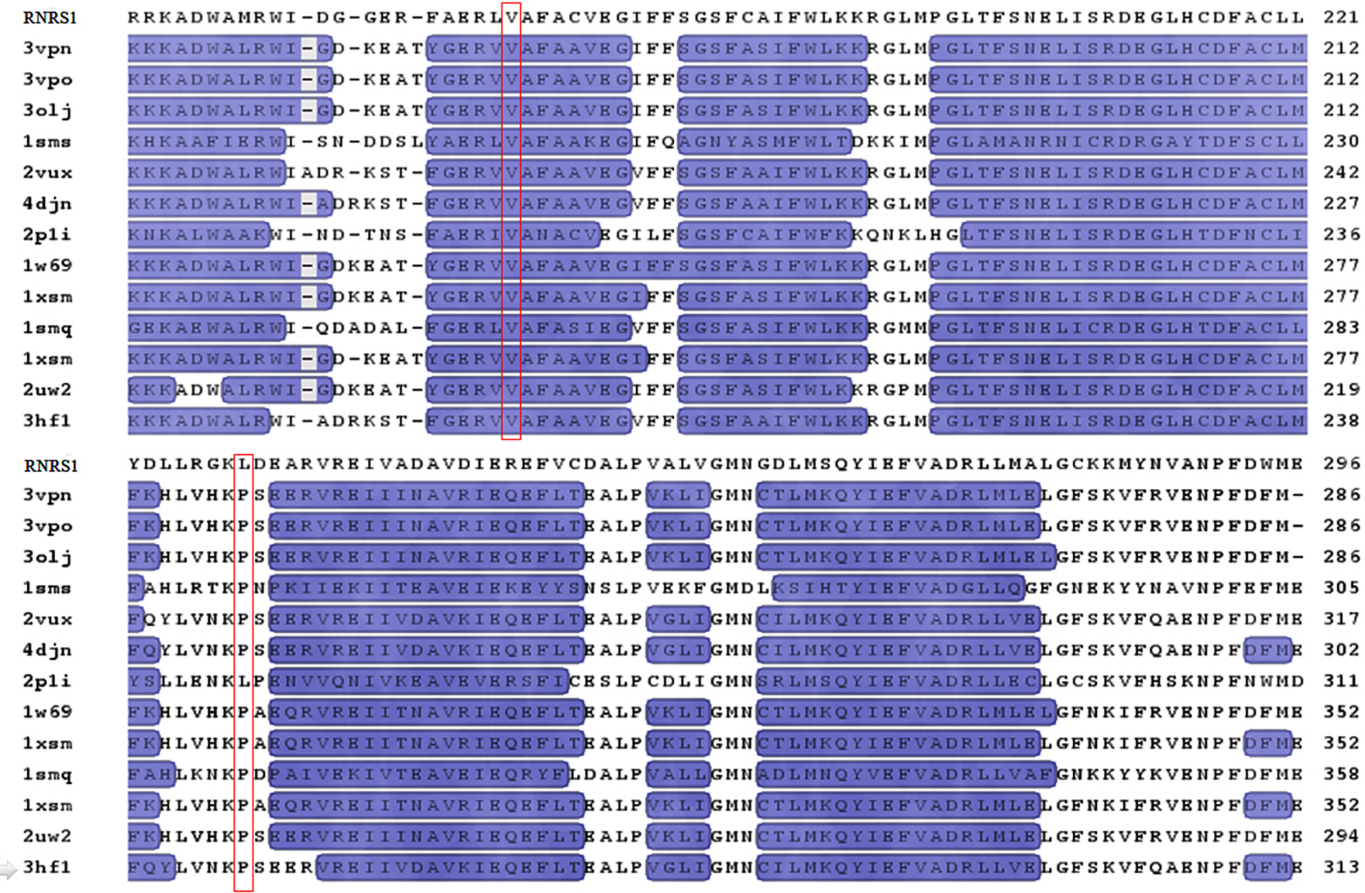
Alignment of the amino acid sequence deduced for RNRS1 and its homologues. The α helix and loop regions were shown in blue and black, respectively. The conserved sites of 171- and 229-amino acid residue are shown in red. Information of reference sequence are as follows: RNRS, small subunit of ribonucleotide reductase 1; 3vpn, A ribonucleoside-diphosphate reductase subunit M2; 3vpo, A ribonucleoside-diphosphate reductase subunit M2; 3olj, A ribonucleoside-diphosphate reductase subunit M2; 1xsm, A ribonucleoside reductase R2; 2vux, A ribonucleoside-diphosphate reductase subunit M2 B; 4djn, A ribonucleoside-diphosphate reductase subunit M2 B; 2p1i, A ribonucleotide reductase, small chain; 1w69, A ribonucleoside-diphosphate reductase M2 chain; 1smq, A ribonucleoside-diphosphate reductase small chain 1; 2uw2, A ribonucleoside-diphosphate reductaseM2 subunit; 3hf1, A ribonucleoside-diphosphate reductase subunit M2 B; 1jk0, A ribonucleoside-diphosphate reductase small chain 1.

The structure of RNRS1 was modeled with p53R2 from Human (PDB:3HF1). The structure consisted of helices and loops (Figs 6 and 7). The iron-binding site is created by α-helices B, C, E, and F [22]. The residue V171 located in the helix 10 corresponds to the helix E in p53R2. Val171 is located in the iron-binding site; thus, the mutation from Val171 to Phe171 in *st1-2* possibly affects the iron-binding site and influences the function. The residue L229 located in the loop determines the functional specificity of a given protein framework [23]. Multiple sequence alignment and homology modeling analysis indicated that V171 is conserved among all the species. However, the residue of L229 is less conserved since this residue is proline in *Saccharomyces cerevisiae*, mouse, and human, but leucine in *Plasmodium yoelii*. Thus, our results show that the two mutants show two different phenotypes and abnormal development of chloroplast comparing to the wild type.

### Expression analysis of genes involved in chlorophyll biosynthesis pathway in mutants and wild type

Our study implicated the missense mutation of RNRS1 in decreasing chlorophyll content in different stages of development. Thus, we used real-time PCR to study the expressions of genes involved in the Chls biosynthetic pathway at different developmental stages (Tables 2, 3 and 4). RNRS1 mainly affected *Porphobilinogen synthase*, *Coproporphyrinogen oxidative decarboxylase*, *Protoporphyrinogen oxidase*, *Chlorophyll synthase*, *Chlorophyllide ‘a’ oxygenase* of (chloroplast biosynthesis pathway) and *pheophorbide a oxygenase* (degradation pathway). The expression of *Porphobilinogen synthase* expression decreased by 21%, 31%, and 43%, in *st1-*2 as compared with WT in all of the three development stages. *Porphobilinogen deaminase* in *st1-*3 decreased 17%, 10%, and 36% compared with WT. *Chlorophyllide a oxygenase* increased as compared with the wild type in the heading stage; however, it was lower as compared with the WT wild in tillering and jointing stage. Therefore, RNRS1 regulates some genes of the chlorophyll biosynthetic pathway, and its mutants show reduced enzyme activity and function in chlorophyll biosynthesis and degradation pathways. *Chlorophyllide a oxygenase* expression increased in the heading stage, and its expression in *st1-3* was 2.47 times than that of wild and expressed order as follows: *st1-3*> *st1-2*>WT. RNRS1 transcripts in WT was about six times than that in *st1-*2 and *st1-*3 in tillering stage but decreased to twice in heading stage because of high temperature.

**Table 2:** Transcription level comparison of gene involved in Chlorophyll biosynthesis pathway between wild type and mutants in tillering stage.

Gene name	WT	*St1-*2	*St1-*3	Compared with WT (%)	
				*St1-*2	*St1-*3
*Porphobilinogen synthase*	1.27±0.40	1.00±0.21	1.06±0.30	-21%	-17%
*Coproporphyrinogen oxidative decarboxylase*	2.53±0.92	0.91±0.25	1.01±0.22	-64%	-60%
*Protoporphyrinogen oxidase*	2.21±0.54	0.84±0.32	1.12±0.33	-62%	-49%
*Chlorophyll synthase*	1.82±0.59	1.00±0.32	0.83±0.21	-45%	-54%
*Chlorophyllide a oxygenase*	2.53±0.89	0.65±0.17	0.63±0.23	-74%	-75%
*Pheophorbideaoxygenase*	1.92±0.84	0.90±0.24	1.12±0.32	-53%	-42%

**Table 3:** Transcription level comparison of gene involved in Chlorophyll biosynthesis pathway between wild type and mutants in jointing stage,

Gene name	WT	*St1-*2	*St1-*3	Compared with WT (%)	
				*St1-*2	*St1-*3
*Porphobilinogen synthase*	1.33±0.51	0.93±0.27	1.20±0.30	-31%	-10%
*Coproporphyrinogen oxidative decarboxylase*	1.70±0.51	0.89±0.20	1.00±0.31	-48%	-41%
*Protoporphyrinogen oxidase*	3.81±1.05	0.69±0.21	0.11±0.04	-82%	-97%
*Chlorophyll synthase*	1.80±0.46	0.94±0.25	1.00±0.34	-48%	-44%
*Chlorophyllide a oxygenase*	1.81±0.62	1.20±0.26	0.73±0.22	-34%	-60%
*Pheophorbideaoxygenase*	3.32±0.75	0.94±0.21	1.00±0.31	-72%	-70%

**Table 4:** Transcription level comparison of gene involved in Chlorophyll biosynthesis pathway between wild type and mutants in heading stage.

Gene name	WT	*St1-*2	*St1-*3	Compared with WT (%)	
				*St1-*2	*St1-*3
*Porphobilinogen synthase*	1.57±0.60	0.89±0.27	1.00±0.43	-43%	-36%
*Coproporphyrinogen oxidative decarboxylase*	1.14±0.36	0.43±0.25	1.00±0.39	-62%	-12%
*Protoporphyrinogen oxidase*	0.89±0.35	0.31±0.11	1.00±0.32	-65%	12%
*Chlorophyll synthase*	2.46±0.63	0.70±0.25	1.00±0.52	-72%	-59%
*Chlorophyllide a oxygenase*	0.26±0.62	0.36±0.21	1.00±0.44	38%	247%
*Pheophorbideaoxygenase*	2.47±0.41	0.45±0.28	1.00±0.39	-82%	-60%

**Fig 8:**
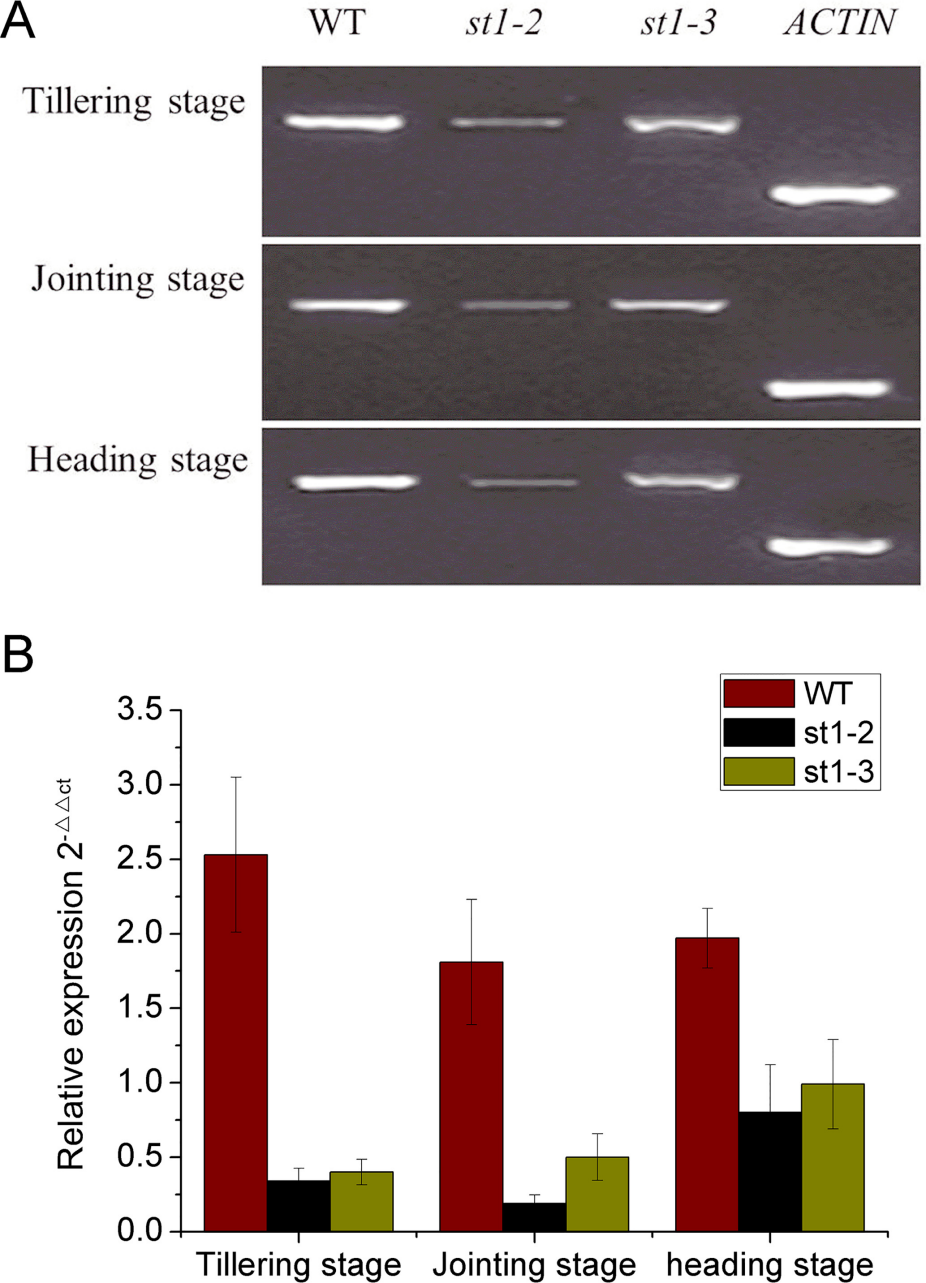
Transcription levels of RNRS1 gene with different development stage among WT and mutants. A, electrophoregram results for expression patterns of RNRS1 gene with different development stages by RT-PCR; B, Transcription levels of RNRS1 gene with different development stages by real-time PCR.

The above results showed that RNRS1 mutants are temperature sensitive and significantly differ from each other in their temperature sensitivities. We noted high expression levels of *RNRS1* gene at different development stages in WT as compared with mutants. However, but *st1-3* and *st1-2* transcripts were not significantly different from each other at any of the developmental stages (Fig 8).

**Fig 9:**
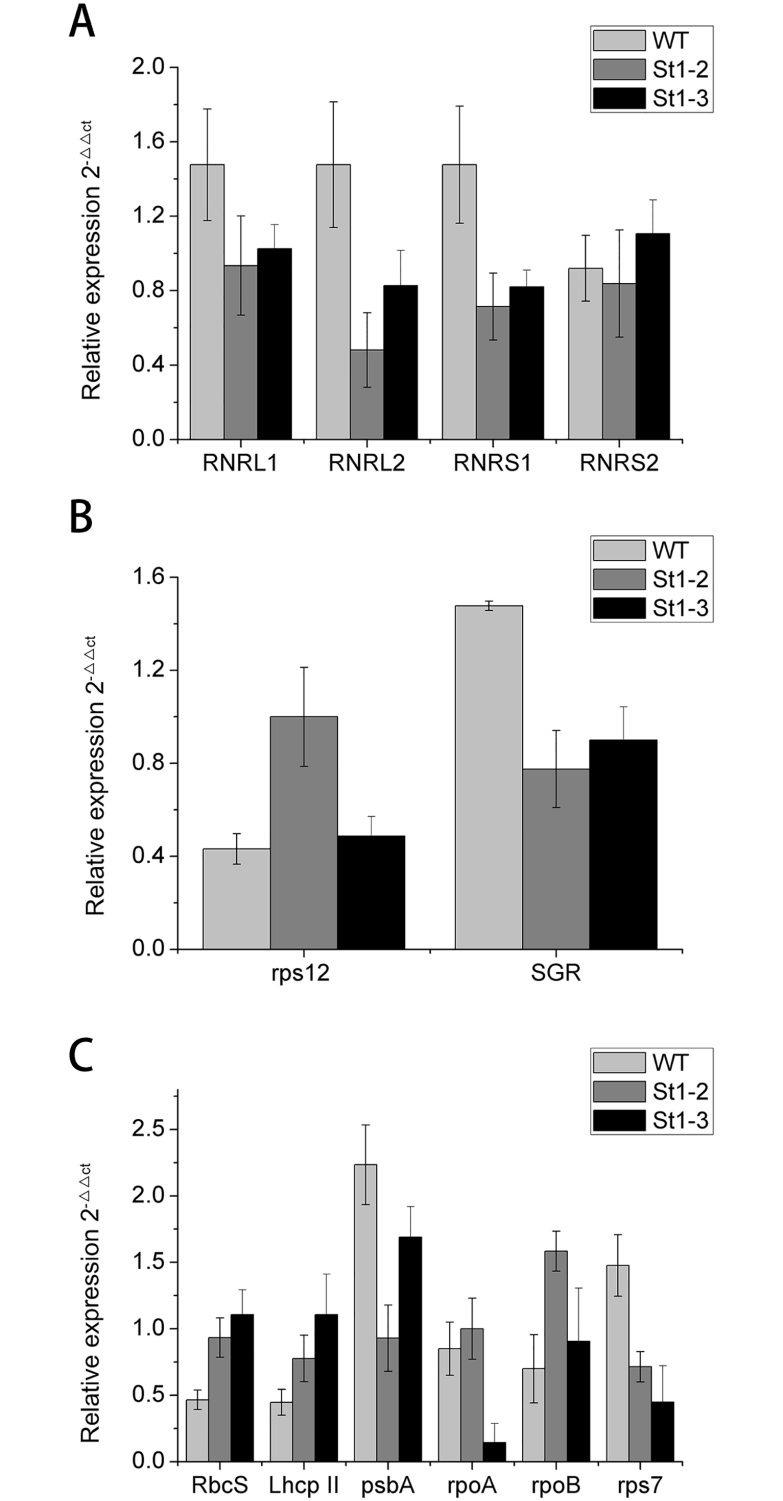
Transcription levels for plastid-nuclear genes involved in chlorophyll biosynthesis affected indirectly in tillering stage between WT and mutants.

Real-time PCR of plastid and nuclear genes in chloroplast biosynthesis revealed the differential expression of these genes in WT and mutants (Fig 9). RNRL1, RNRL2, and RNRS1 showed higher expression levels in WT as compared with mutants and showed the following trend:WT> *st1-3*> *st1-2*. However, RNRS2 (small subunits of ribonucleotide reductase) expression differed in *st1-2* and *st1-3*, and its expression content in *st1-3* was significantly higher than that in WT with *st1-3*> WT> *st1-2*. RNRS1 probably suppresses RNRL expression in the two mutants owing to the missense mutation. On the contrary, RNRS1 in *st1-3* contributes to *RNRS2* transcription because of a missense mutation, C685T, Leu to Phe.

Chloroplast development requires the precise coordination of gene expression and is via a two-way signal between plastid and nuclear genes. We analyzed the differences in copy numbers of chloroplast DNA relative to nuclear DNA between wild type and mutants using real-time PCR. For this we used the *Stay-green* (*SGR*; Park et al. [24]) and *rps12* (Sugimoto et al. [13]) mutants, which exist as single-copy genes in the nuclear and plastid genomes, respectively (Fig 9B). Our results showed that RNRS1 mutant negatively regulates nuclear gene (*SGR*) and showed almost 50% lower expression than WT. By contrast, the plastid gene (*rps12*) was positively regulated and showed over 50% elevated expression in WT. Moreover, our results revealed that *rps12*-*SGR* showed suppression relationship with each other as Yoo et al. [9] previously reported.

We further investigated whether the reduced activity of RNRS1 in *st1-2* and *st1-3* affected nuclear and plastid genes in the tillering stage (Fig 9C). The genes *psbA* and *rps7* showed higher expression in WT than in *st1-2* and *st1-3*. However, other genes such as *RbcS*, *Lhcp*II, *rpoA* and *rpoB* showed lower expression in WT as compared with that of *st1-2* and *st1-3*. Our results indicate that RNRS1 mutant is only partially involved in photosynthetic and plastid gene transcription apparatus.

### Expression analysis of chloroplast development genes in mutants in response to different temperatures

**Fig 10:**
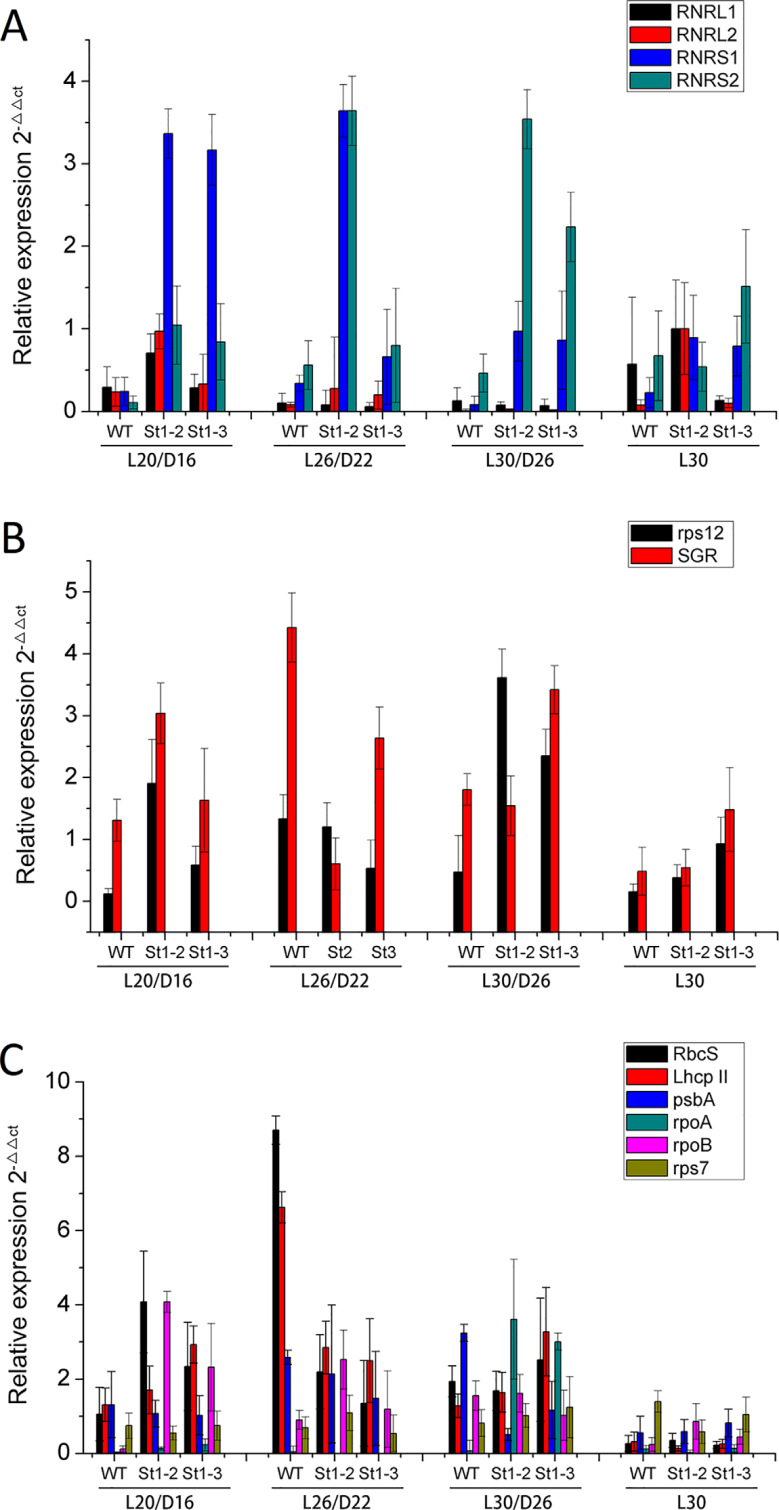
Transcription analysis for plastid-nuclear genes involved in chlorophyll biosynthesis affected indirectly between WT and mutants by different temperature dealt. L expresses light condition; D expresses dark condition.

The temperature sensitivity of different mutants was studied by growing the mutants in different temperature conditions. Both *st1-2* and *st1-3* all showed white stripes starting at stage L2. We compared the mutant plant growth with that of wild type before conducting further experiments. The mutants *st1-*2 and *st1-*3 showed retarded growth throughout their development (Fig 1C and 1D). Temperature studies in RNR showed that the expression of two smaller subunits was highly variable in response to temperature changes as compared with larger subunits (Fig 10A). The small subunits RNRS1 were up-regulated ten times in *st1-2* plants at L20/D16, L26/D22 and L30/D26 as compared with WT (Fig 10A). By contrast, RNRS2 in *st1-2* mainly was significantly up-regulated at L26/D22 and L30/D26 as compared with WT. However, expression of RNRS1 subunit in *st1-3* was significantly less than in *st1-2*, but the expression of RNRS2 subunit was higher than that in *st1-2*. Therefore, we speculate that the two different mutant locations were responsible for the differential expression of the two small subunits.

**Fig 11:**
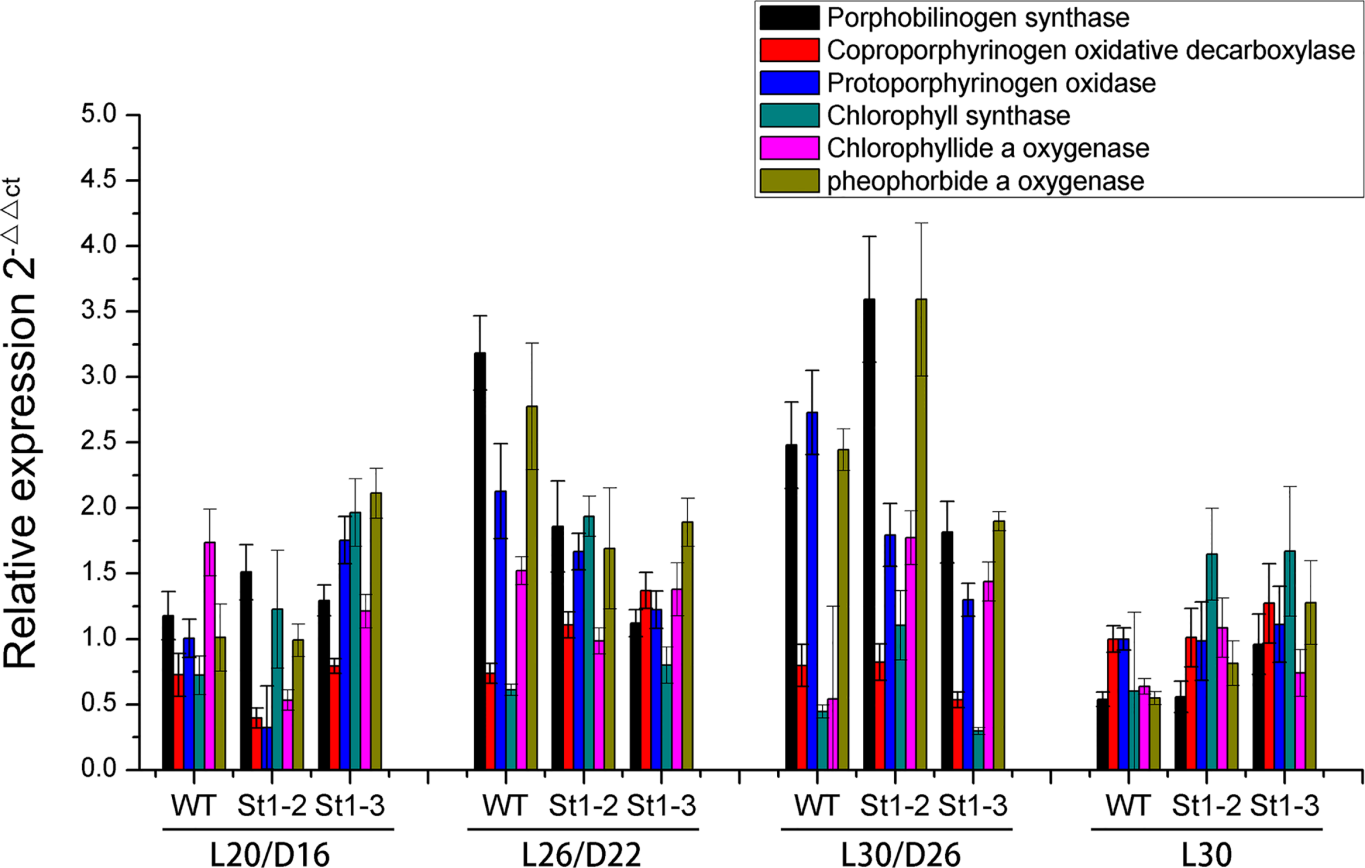
Transcription analysis of gene of chlorophyll biosynthesis affected directly between WT and mutants by different temperature dealt. L expresses light condition, D expresses dark condition.

We analyzed the differences in the copy numbers of chloroplast DNA relative to nuclear DNA between WT and mutant types using real-time PCR with *Stay-green* (*SGR*; Park et al. [24]) and *rps12* [13], which exist as single-copy genes in the rice nuclear and plastid genomes, respectively (Fig 10B). The expression patterns of mutants were different from *rps12* for different temperature treatments. *SGR* mutants showed significantly higher expression as compared to WT in the following conditions: L20/D16, L30/D26, and L30. The expression patterns of *rps12* were similar to *SGR* in both mutants and WT. However, *SGR* and *rps12* expression levels were significantly higher in WT than mutant in L26/22 indicating that temperature effects both the mutants and WT. Chloroplast development during leaf development is tightly regulated by the coordinated expression of plastid and nuclear genes such as plastid genes *psbA*, and nuclear genes *Rbcs* and *LhcpII* (Mandel et al., [1] (Fig 10C). The transcripts of photosynthetic genes encoded in the plastid *psbA* were higher in WT than mutants in different treatments (L20/D16, L26/D22 and L30/26); *st1-3* showed a slight increase in response to L30. The nuclear genes *Rbcs* and *LhcpII* were significantly difference among WT and mutants in different temperature conditions (Fig 10). *Rbcs* and *Lhcp* transcripts were significantly higher in mutants than WT during L20/D16 treatment. However, WT showed higher transcript levels of *Rbcs* and *LhcpII* at L26/D22 as compared with *st1-2* and *st1-3*. Similar to *Rbcs* and *Lhcp* genes, *rpoB* transcripts varied significantly between WT and mutants. It was up-regulated in mutants at L20/D16 as compared with WT. On the other hand, the transcript levels of the plastid gene encoding the transcription/translation apparatus (*rpoA*) was barely detectable. The transcripts of the plastid gene *rps7* did not differ significantly among WT and mutants. Therefore, RNRS1 regulates only some of the genes involved in photosynthesis. Moreover, *Porphobilinogen synthase*, *Chlorophyll synthase*, and *pheophorbide a oxygenase* for transcripts were significantly affected by temperature changes in both WT and mutants (Fig 11).

## Discussion

Chloroplast mutants such as *v1*, *v2*, *v3* and *st1* are sensitive to temperature and show varied phenotypes [9,10,13]. Kusumi et al. [25] reported temperature sensitivity caused the zebra leaf phenotype in chloroplast mutants. *Young leaf chlorosis 1* mutant displays abnormal leaf color in the early development stage, and normal green color with an increase in temperature [26]. Moreover, different mutants show different degrees of sensitivities to temperatures, for example: *v3* encoding mutant of RNRL1 is more sensitive to temperature than *st1* encoding RNRS1. Our study showed that rice *stripe1- 3* mutant (*st1-*3) is more sensitive to temperature than rice *stripe 1–2* mutant (*st1-*2). Moreover, the two mutants showed different phenotypes, for example; *st1-2* presented conspicuous white stripe leaves as compared with *st1-3* owing to the different mutant location associated with encoding RNRS1. The mutants, *st1*, *st1-*2 and *st1-*3 associated with RNRS1 are sensitive to temperature and show different phenotypes. Therefore, we speculate that the genes involved in chloroplast development in rice are mainly affected by temperature despite being differentially regulated. Our study further proves that both large and small subunits of ribonucleotide reductase regulate leaf color phenotypes during chloroplast development. Yoo et al. (2009) reported that *st1* encoded RNRS1 and displayed white-stripe phenomenon in the early developmental stage, which reverts to normal green leaf phenotype with an increase in permissive temperature. Similarly, *st1-3* reverts to normal leaf phenotype at 30°C. However, *st1-2* could not produce normal green colored leaves even at a permissive temperature as compared with *st1* and *st1-3* [9]. RNRS1 contains only one conserved domain (amino acids 25–300), and the Arg-40 residue of *st1* is conserved as Lys in all living organisms [9]. However, our study on chloroplast development in rice showed that Phe-171 residues of *st1-2* and Phe-229 residues of *st1-3* are conserved as Val and Leu, respectively. Besides, the mutation located on 171 (Val to Phe residue) affects chloroplast development more than that on 229 (Leu-Phe residues). The missense mutation resulting in *st1-2* strongly suppresses chloroplast development and confers lower temperature sensitivity in *st1-*2 as compared with *st1* and *st1-3*. We speculate that the mutation in the iron-binding site (from Val171 to Phe171) and α helix activity region located in *st1-2* may affect iron chelation activity; thus, influencing chloroplast development. Elucidation of protein structure would provide new insights into the conserved RNRS1 domain.

The *zebra* mutant of rice shows disorganized thylakoid membrane system [25], small osmiophilic plastoglobules, and many crystalloids. The *ylc1* gene mutant affects chlorophyll and lutein development and shows loose thylakoid lamellar structure in young leaves [26]. The mutant *st1-2* also shows loose thylakoid lamellar structure during late leaf development. Contrastingly, *st1* shows normal thylakoid lamellar structure [9] as observed in *st1-3* in our study. Whether, the genes affecting chloroplast development also regulate the structural abnormalities in thylakoid lamellae is yet to be elucidated. The mutant *st1-2* affects plant development, photosynthesis, and rice yield more than *st1-3*. Real-time PCR studies showed that RNRS1 was significantly higher in WT, following *st1-3* and *st1-2* in all developmental stages. The genes involved in chloroplast biosynthesis and degradation pathways were down-regulated in all stages of development. Thus, it is reasonable to deduce that RNRS1 controls chloroplast development. The phenotype *st1-2* shows a mutation in the α helix activity region that affects the binding site, due to which it regulates the genes of chloroplast biosynthetic pathway more than *st1* and *st1-3*. The identification of the mutation site also revealed the iron-binding site in these mutants. RNRL1 and RNRS1 are significantly up-regulated in *st1* mutants regardless of the temperature conditions, whereas there is only a slight increase in the expression of RNRL2 [9]. RNRS2 expression was almost unaltered as compared with WT. RNRL1 and RNRL2 transcripts in *st1-2* and *st1-3* showed a slight increase increased in L20/D16 as compared to WT. RNRS1 and RNRS2 were both significantly up-regulated at L20/D16, L26/D22 and L30/D26 as compared with WT. Perhaps, the change from aliphatic amino acid to aromatic amino acids caused the slight change in protein spatial structure and eventually affected the link between α_1_ and β_1_, and α_2_ and β_2_.

RNRL1 mutants show virescent leaf, RNRS1 display stripe leaf, and both mutants are temperature-sensitive. RNRS1 in *st1* influences the transcript levels of photosynthetic genes (*psbA*) and nucleus (*RbcS* and *LhcpII*)). On the contrary, *rpoA*, *rpoB*, and *rps7* are positively regulated in the *st1* mutant leaf. Similar to the expression of *RbcS*, and *LhcpII in st1*, the expression of *RbcS* and *Lhcp* in *st1-2* and *st1-3* was higher than WT [9]. However, the expression of *psbA* in WT was significantly higher than that in *st1-2* and *st1-3*. Our results showed that RNRL1 and RNRS1 are more up-regulated at 20°C than at 30°C. The small subunits for transcript levels were significantly up-regulated in *st1-2* and *st1-3* than the large subunits. Yoo et al. reported that RNRL1 in *v1* and RNRS1 in *st1* are weak mutations in terms of RNR activity [9]. However, RNRS1 mutant in *st1-2* was a stronger mutation than those in *st1-3* and *st1*. Thus, *st1-3* influences the transcript genes involved in chloroplast development as a result of a mutation in Leu-229 residues locate loop region. Besides, missense mutant for *β*_*1*_ in *st1-2* causes changes in enzyme structure affecting the linkage of *α1* and β1. Protein crystallography studies of RNRS1 protein will provide deeper insights into its spatial structure.

## Supporting Information

S1 Fig Alignment of DNA (A) and protein (B) sequences between WT and *st1-3*. (TIF)

S2 Fig Phenotypic characteristics of reciprocal cross in between *st1-2* and *st1-3*. A, Phenotype characteristics of reciprocal cross at the younger stage; B, Phenotypes characteristics of reciprocal cross at the heading stage.
(TIF)
