# Supplementary figures and images for: Rice stripe1-2 and stripe1-3 Mutants Encoding the Small Subunit of Ribonucleotide Reductase Are Temperature Sensitive and Are Required for Chlorophyll Biosynthesis
Source: PLoS One. 2015 Jun 23;10(6):e0130172. doi: 10.1371/journal.pone.0130172 (PMC4478038; doi:10.1371/journal.pone.0130172)

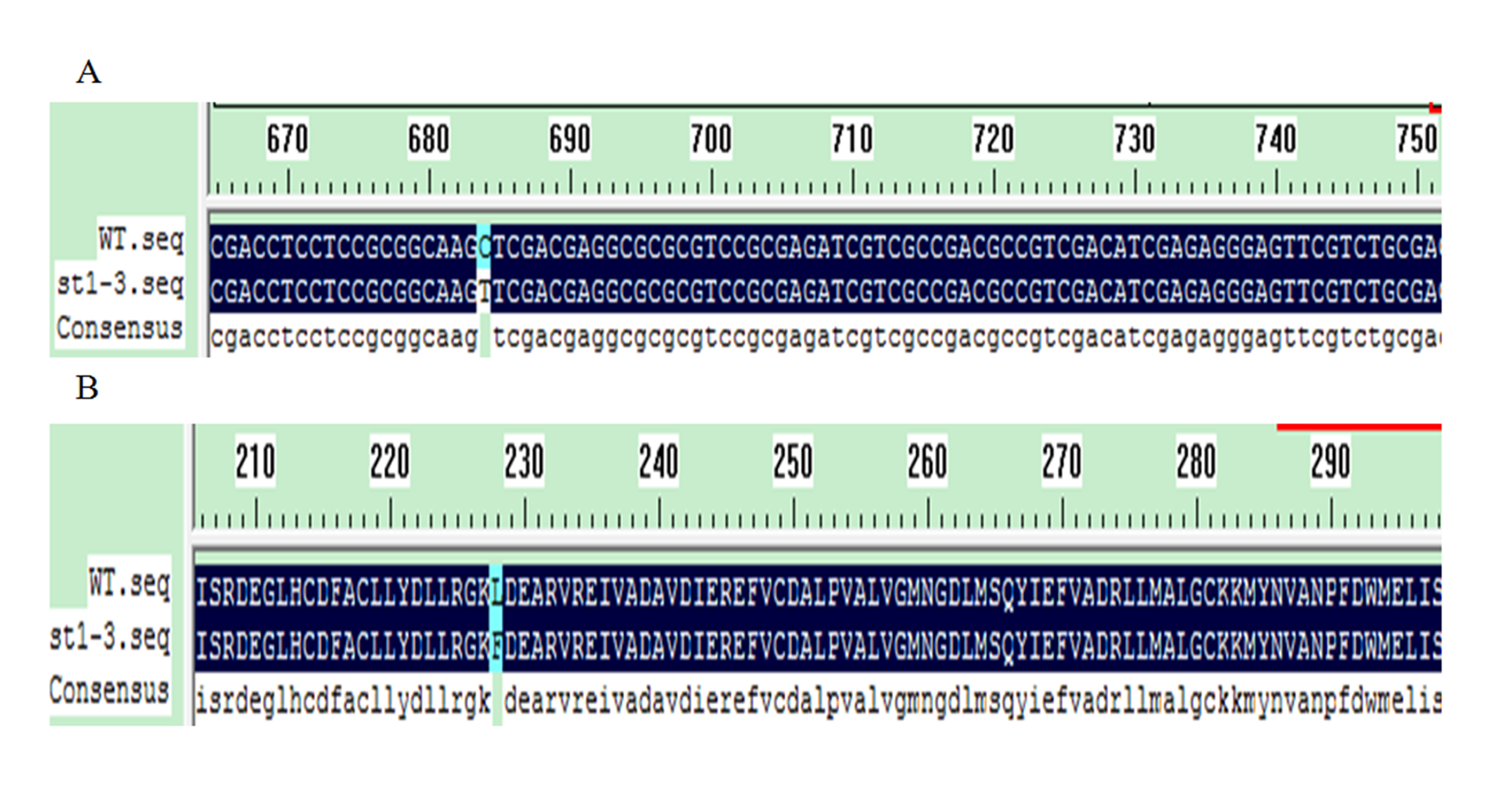

Supplement: S1 Fig — (TIF) [file pone.0130172.s001.tif]

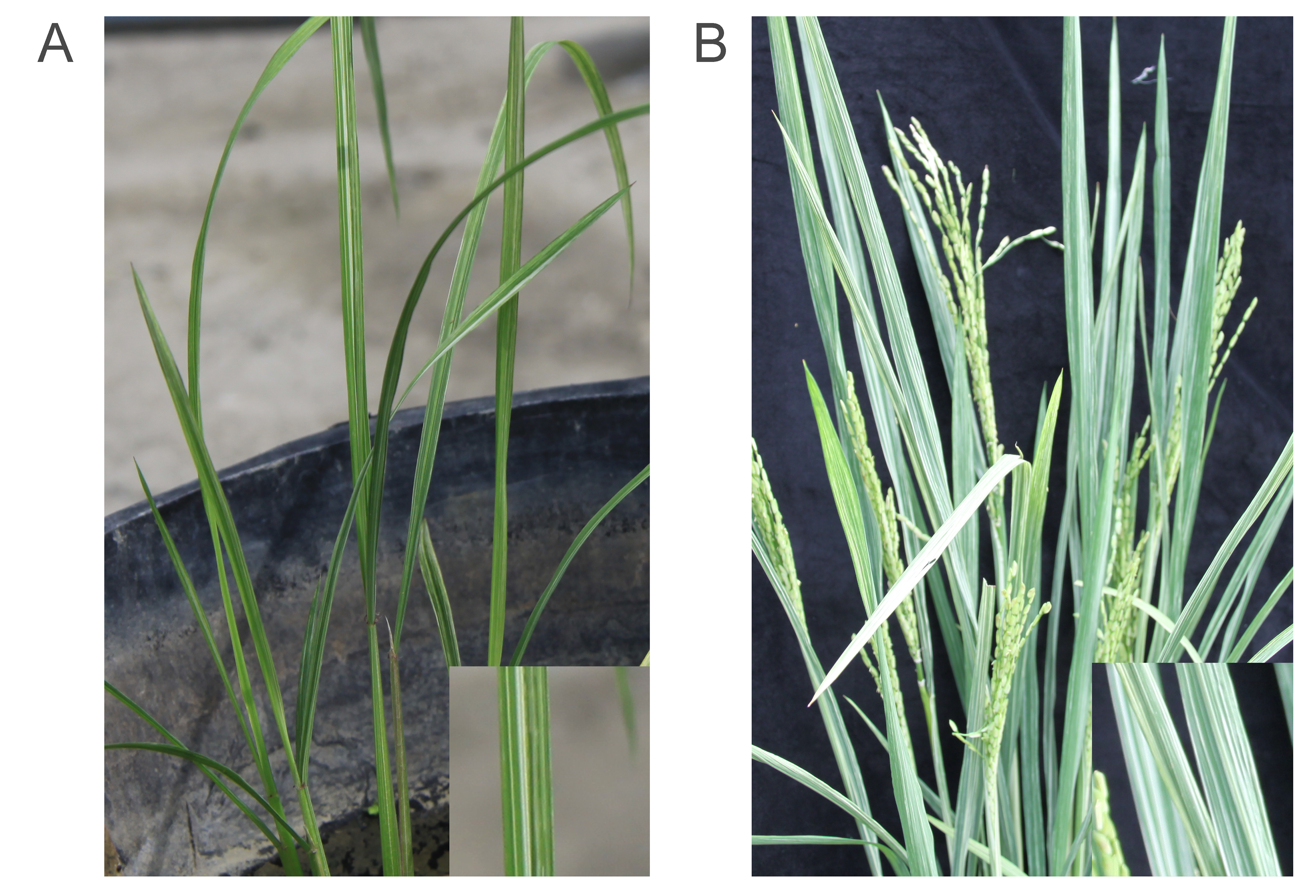

Supplement: S2 Fig — A, Phenotype characteristics of reciprocal cross at the younger stage; B, Phenotypes characteristics of reciprocal cross at the heading stage. (TIF) [file pone.0130172.s002.tif]
